# A Novel Antithrombotic Protease from Marine Worm *Sipunculus Nudus*

**DOI:** 10.3390/ijms19103023

**Published:** 2018-10-04

**Authors:** Ya-Hui Ge, Yan-Yan Chen, Gui-Sheng Zhou, Xin Liu, Yu-Ping Tang, Rui Liu, Pei Liu, Na Li, Jie Yang, Jing Wang, Shi-Jun Yue, Huiping Zhou, Jin-Ao Duan

**Affiliations:** 1 Key Laboratory of Shaanxi Administration of Traditional Chinese Medicine for TCM Compatibility, and Shaanxi Key Laboratory of Chinese Medicine Fundamentals and New Drugs Research, and Shaanxi Collaborative Innovation Center of Chinese Medicinal Resources Industrialization, Shaanxi University of Chinese Medicine, Xi’an 712046, China; geyahui_jsnt@163.com (Y.-H.G.); chenyanyan59@163.com (Y.-Y.C.); 1351013@sntcm.edu.cn (J.Y.); wangjing19890126@126.com (J.W.); shijun_yue@163.com (S.-J.Y.); 2 Jiangsu Collaborative Innovation Center of Chinese Medicinal Resources Industrialization, and National and Local Collaborative Engineering Center of Chinese Medicinal Resources Industrialization and Formulae Innovative Medicine, and Jiangsu Key Laboratory for High Technology Research of TCM Formulae, Nanjing University of Chinese Medicine, Nanjing 210023, China; zhouguisheng1@126.com (G.-S.Z.); liuxin_njutcm@163.com (X.L.); liurui@njucm.edu.cn (R.L.); liupei@njucm.edu.cn (P.L.); dja@njucm.edu.cn (J.-A.D.); 3 State Key Laboratory of Quality Research in Chinese Medicine, Macau Institute for Applied Research in Medicine and Health, Macau University of Science and Technology, Avenida Wai Long, Taipa 999078, Macau, China; nli@must.edu.mo; 4 Department of Microbiology and Immunology, Virginia Commonwealth University, Richmond, VA 23298, USA; Huiping.Zhou@vcuhealth.org

**Keywords:** *Sipunculus nudus*, isolation and purification, fibrinolytic activity, antithrombotic activity, metabonomics

## Abstract

*Sipunculus nudus*, an old marine species, has great potential for use as functional seafood due to its various bioactivities. Its potential antithrombotic activity pushed us to isolate the bio-active components bio-guided by tracking fibrinolytic activity. As a result, a novel protease named as SK (the kinase obtained from *S. nudus*) was obtained, which possessed a molecular weight of 28,003.67 Da and 15 N-terminal amino acid sequences of PFPVPDPFVWDTSFQ. SK exerted inhibitory effects on thrombus formation through improving the coagulation system with dose-effect relationship within a certain range. Furthermore, in most cases SK got obviously better effect than that of urokinase. With the help of untargeted mass spectrometry-based metabolomics profiling, arachidonic acid, sphingolipid, and nicotinate and nicotinamide mechanism pathways were found to be important pathways. They revealed that the effect mechanism of SK on common carotid arterial thrombosis induced by FeCl_3_ was achieved by inhibiting vessel contraction, platelet aggregation, adhesion, and release, correcting endothelial cell dysfunction and retarding process of thrombus formation. This study demonstrated SK was a promising thrombolytic agent on the basis of its comprehensive activities on thrombosis, and it should get further exploitation and utilization.

## 1. Introduction

Thrombotic disorders, including ischemic stroke, unstable angina, pulmonary embolism (PE), deep venous thrombosis (DVT) and myocardial infarction, are major causes of morbidity and mortality worldwide [1]. A number of antithrombotic agents, which include anticoagulant, antiplatelet, and fibrinolytic agents, are available for the treatment and prevention of thrombosis. Warfarin is the first widely used anticoagulant shortly followed by heparin. Heparin, which is the second-most used naturally occurring drug in medicine, is widely applied in clinical for a long term. But, the long-standing use of anticoagulant can cause a number of side effects, such as bleeding, thrombocytopenia, changes in lipid metabolism, and osteoporosis [2,3,4]. Subsequently, the antithrombotic agents, such as nattokinase, streptokinase, urokinase, and t-PA are exploited for treatment of thrombosis and they show significant therapeutic effects [5,6]. However, these antithrombotic agents are very expensive and still present some inescapable side effects [5,6,7]. Hence, the existing antithrombotic agents cannot satisfy the requirements of patients. Due to the value of antithrombotic agents, the search for other antithrombotic agents has been implemented from various sources without interruption since the 1990s. Increasing studies indicate that many new thrombolytic agents are identified and characterized from natural resources, such as *Eisenia foetida*, snake venoms, *Neanthes japonica*, *Urechis unicinctus,* and so on [8,9,10].

*Sipunculus nudus*, an old marine species, approximately emerged in Middle Paleozoic Era and even Cambrian Period. *S. nudus* belongs to the *Sipuncula* phylum and it has a wide distribution along the coast of Indian Ocean and Western Pacific [11]. *S. nudus* is an edible marine organism that is popular for its delicious taste in southeast China for many years. With the help of modern technology, it is found that *S. nudus* has great potential for use as functional seafood due to its various bioactivities, such as anti-thrombus [12], anti-hypoxic, anti-fatigue [13], immune modulatory [14], antimicrobial, and antioxidant activities [15,16]. Recently, the potential antithrombotic activity of *S. nudus* has attracted considerable attention, as it is considered to be rich in free amino acids, polypeptides, and proteins. By the inspiration of obtaining multiple fibrinoclase fromnatural resources as well as consideration of close affinity among *Sipuncula*, *Annelida,* and *Echiura* [17], the antithrombotic activity of aqueous extract from *S. nudus* was evaluated in our previous work, and the results indicated that the crude protein extract of *S. nudus* had strong activities of fibrinolysis, anti-coagulating, inhibiting platelet aggregation, as well as scavenging oxygen free radical, and so on [12]. Accordingly, to find out the bio-active ingredients, the present study reported the isolation, purification, and characterization of a novel protease named SK (the kinase obtained from *S. nudus*) from *S. nudus* and its antithrombotic effects, as well as the related mechanism.

## 2. Results

### 2.1. The Preparation, Isolation and Purification of SK

The contents of samples were displayed in Table S1. From the result, the yield of crude extract was relatively high and approximate to 4%.

**Figure 1:**
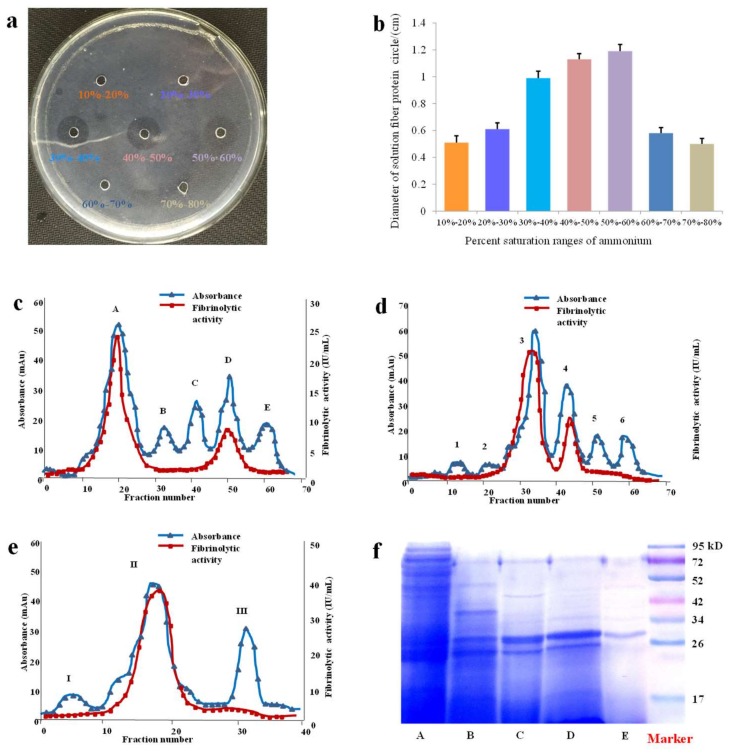
Fibrinolytic activity of protein precipitated by different percent saturation ranges of (NH_4_)_2_SO_4_ and purification of SK. (**a**) Fibrin plates. (**b**) The diameter of fibrinolytic circle affected by protein extract precipitated with 10–20%, 20–30%, 30–40%, 40–50%, 50–60%, 60–70%, and 70–80% saturation ammonium sulfate (**c**) Ultraviolet absorbance and fibrinolytic activity profile of Phenyl Sepharose High Performanc (**d**) Ultraviolet absorbance and fibrinolytic activity profile of Q Sepharose High Performance. (**e**) Ultraviolet absorbance and fibrinolytic activity profile of Superdex prep grade G-75. (**f**) Electronic analysis of cropped SDS-PAGE for different steps, by extraction (A), (NH_4_)_2_SO_4_ precipitation (B), hydrophobic interaction chromatography (C), ion-exchange column chromatography (D) and gel filtration chromatography (E), and full-length gel is presented in Figure S9.

According to percent saturations of (NH_4_)_2_SO_4_ set in the experiment, seven crude protein precipitations were obtained. Evaluation of fibrinolytic activities indicated that protein precipitations in the ranges of 30–40%, 40–50%, and 50–60% saturation showed higher activities than those in the ranges of 10–20%, 20–30%, 60–70%, and 70–80% saturation. These results implied that SK started to subside largely at the beginning of 30% saturations and had mainly precipitated till 60% saturations. The related results were shown in Figure 1a,b. Consequently, (NH_4_)_2_SO_4_ of 30–60% saturation was chosen to salt out the active proteins from crude extract of *S. nudus*. After the graded precipitation while using (NH_4_)_2_SO_4_, the crude protein was further purified by hydrophobic interaction chromatography that is based on its hydrophobicity. As shown in Figure 1c, peak A and D exhibited higher fibrinolytic activities as well as protein contents than other peaks, so the fractions of peak A and D were collected and pooled for the next purification. The above-pooled fractions were further purified on the basis of their binding capacities by use of anion-exchange chromatography. From Figure 1d, peak 3 and 4 showed higher fibrinolytic activity and protein content. Hence, peak 3 and 4 were collected and pooled for further purification. Then, gel filtration chromatography was employed to purify the above pooled fractions (peak 3 and 4) based on the difference of molecular weight. The results indicated that peak II possessed higher fibrinolytic activity and protein contents (Figure 1e). In this study, peak II was collected and further purified by Millipore Amicon Ultra 3K ultrafiltration and followed by Zeba spin desalting columns for desalination once more. Finally, the purified peak II as protease SK was obtained and stored at −20 °C for further study.

Electrophoretic analysis of fractions was also carried out on SDS-PAGE for exhibiting the purity of the samples more intuitively by different prepared methods, including direct extraction, (NH_4_)_2_SO_4_ graded precipitation, hydrophobic interaction chromatography, ion exchange chromatography, and gel filtration chromatography (Figure 1f).

### 2.2. The Identification of the Purity, Molecular Weight and N-terminal Amino Acids Sequence of SK

**Figure 2:**
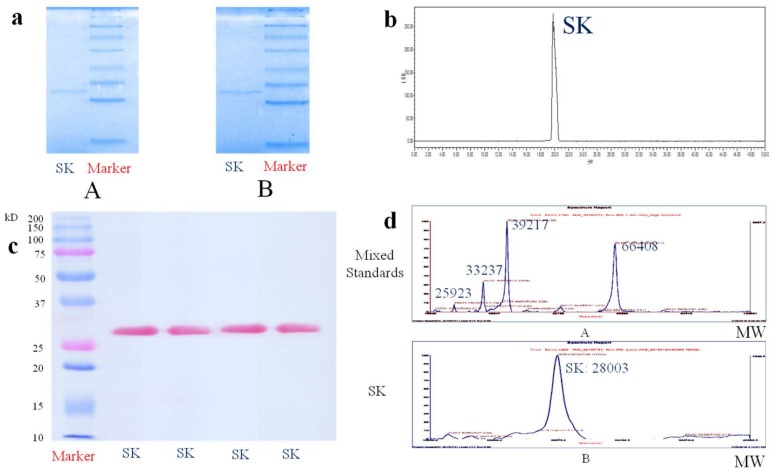
Purity identification of SK. (**a**) The Native–PAGE profile (A) and SDS-PAGE profile (B) of SK. (**b**) Gel filtration chromatogram of SK by UPLC analysis. (**c**) Electroblotting profile of SK. (**d**) The chromatogram of mixed standards with different molecule weight (A) and SK (B) by MALDI-TOF analysis.

In this experiment, the combination of electrophoresis and gel filtration chromatography was used to identify the purity of SK. Since SDS-PAGE was one of the most common approaches to determine the purity of protein, and the separation principle was merely based on the molecular weight. Using the method of SDS–PAGE, the detected protein was firstly denatured by SDS and disaggregated by β-mercaptoethanol to distinguish the protein with different free amino acid composition, but with the same molecular weight. Additionally, the Native PAGE analysis without SDS and β-mercaptoethanol was also used to identify the purity of SK. The results of the above gel electrophoresis analysis showed that SK presented single diffuse bands of 28.0 kDa in both 5% stacking gel and 15% separating gel either under reductive or non-reductive circumstances. The related data were shown in Figure 2a. In addition, the purity of SK was further identified by gel filtration chromatography, which was a mild and rapid method with separation principle of molecular weight, and SK showed a single peak at 20 min in chromatogram (Figure 2b). Consequently, SK was regarded as electrophoretic purity and chromatographic purity while using the methods of SDS–PAGE, Native PAGE, and gel filtration chromatography.

As for the molecular weight determination of protein, since protein possessed large molecular weight with complicated construction and it was hard to ionize. From the previous reports, matrix assisted laser desorption/ionization (MALDI) reflected more advantages over other ion sources [18]. The molecular weight determination of SK premised on the idea that the target plate was firstly calibrated by standards, and the calibration and test results were displayed in Figure 2d. The analytical result of MALDI-TOF indicated that the molecular weight of SK was 28,003.67 Da.

Edman degradation was a classical and powerful technique for analysis of N terminal. During the process, the band of SK in a polyvinylidene difluoride (PVDF) membrane was firstly stained by ponceaux, which also reconfirmed that SK had a single diffuse band of 28,000 Da. The relational electrical graph was shown in Figure 2c. Under the same condition of calibration, 15 N-terminal amino acids sequence of SK was measured as NH_2_-Pro-Phe-Pro-Val-Pro-Asp-Pro-Phe-Val-Trp-Asp-Thr-Ser-Phe-Gln (PFPVPDPFVWDTSFQ) by the method of Edman degradation.

### 2.3. The Enzymatic Properties of SK

#### 2.3.1. Effects of Temperatures on SK

**Figure 3:**
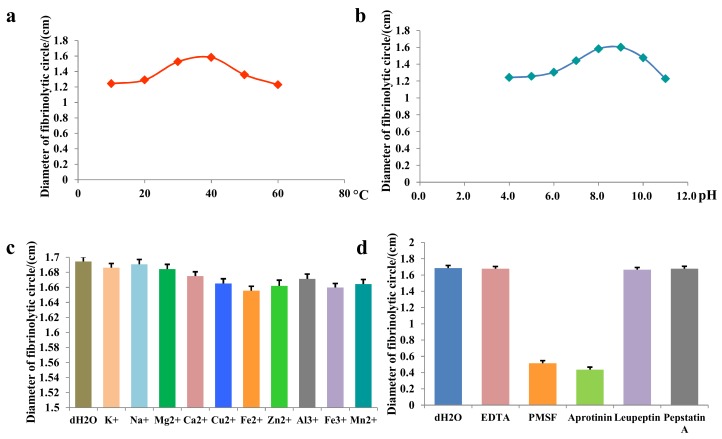
The enzymatic properties of SK. Effects of (**a**) temperature: 10, 20, 30, 40, 50, 60 °C, (**b**) pH: 4, 5, 6, 7, 8, 9, 10, 11, (**c**) metal ions: K^+^, Na^+^, Mg^2+^, Ca^2+^, Cu^2+^, Fe^2+^, Zn^2+^, Al^3+^, Fe^3+^, Mn^2+^ (**d**) inhibitors: EDTA, PMSF, Aprotinin, leupeptin, pepstatin A on fibrinolytic activities of SK.

SK showed temperature–activity relationship in the range 10–30 °C with a notable jump. At 30 and 40 °C, SK showed the strongest residual activities with a slightly increase, nevertheless, the activity of SK decreased sharply at first, and then displayed a decreasing tendency between 40 and 60 °C (Figure 3a).

#### 2.3.2. Effects of pH Values on SK

For the study on the effects of pH values, Figure 3b showed that the residual activities of SK shot up with increasing pH values in the range 4–8, and the activities reached to maximum activity between pH 8 and 9. As pH increasing in the range 9–11, the activities gradually decreased and reached minimum activity at pH 11.

#### 2.3.3. Effects of Metal Ions on SK

By the use of mixture of deionizedwater and SK as control, the effects of 10 metal ions (K^+^, Na^+^, Mg^2+^, Ca^2+^, Cu^2+^, Fe^2+^, Zn^2+^, Al^3+^, Fe^3+^, and Mn^2+^) on SK were not significant with great differences. Among the metal ions, Fe^2+^ had the highest inhibitory effect, while Na^+^ had the lowest inhibitory effect (Figure 3c).

#### 2.3.4. Effects of Inhibitors on SK

Contracting with mixture of deionizedwater and SK, the inhibitory effects of phenyl methyl sulfonyl fluoride (PMSF)and aprotinin on SK were significant; however, ethylenediamine-tetraacetic acid (EDTA), leupeptin and pepstatin A almost showed no inhibitory effects which were equal to deionized water (Figure 3d).

### 2.4. Fibrinolytic Activity and Fibrinogenolytic Activity of SK

**Figure 4:**
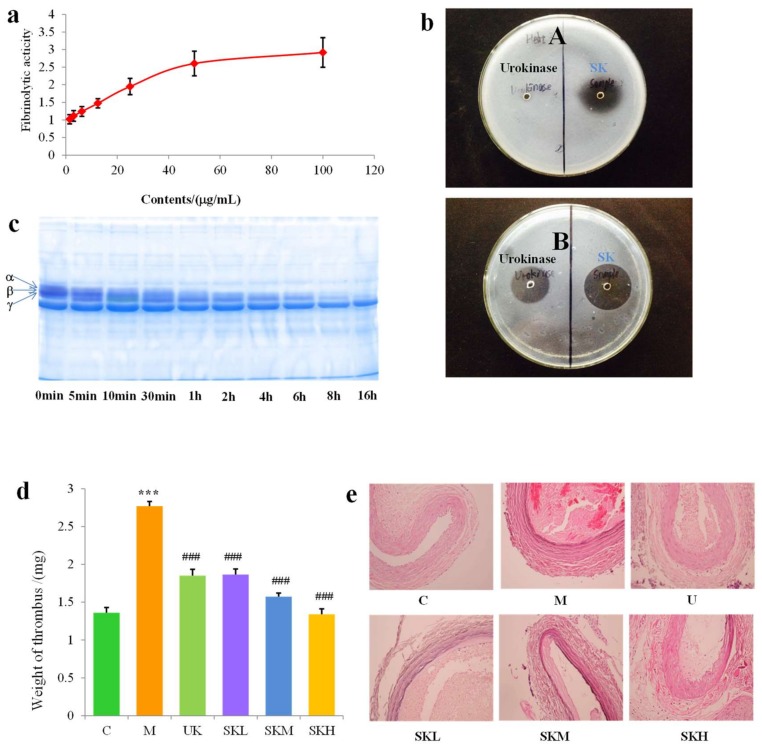
Fibrinolytic activity, fibrinogenolytic activity, and effects on FeCl_3_-induced common carotid arterial thrombosis rat of SK. (**a**) The fibrinolytic activities of SK with different concentration. (**b**) The fibrinolytic activities of urokinase and SK in both plasminogen-free (A) and plasminogen-rich (B) fibrin plates. (**c**) Structure of fibrinogen after effect of SK for different time, and α, β and γ were defined as three polypeptide chanins α, β and γ. (**d**) Effects of SK on the common carotid artrial thrombus weight in rats. *** *p* < 0.001 vs. Sham-operated group; ^###^ *p* < 0.001 vs. Model group. (**e**) Effects of SK on histomorphology of common carotid arterial thrombus in rats (HE, ×200).

Evaluation of in vitro fibrinolytic activities of SK by fibrin plate assay showed that SK had obvious effects of fibrinolysis and the average vertical diameters of fibrinolytic circles in a concentration-dependent manner within the concentration range of 1.53–100.00 μg/mL. In the range of 1.53–60.00 μg/mL, the fibrinolytic activities surged with the increasing of concentration, and in the range of 60–100.00 μg/mL, the fibrinolytic activities went up slowly (Figure 4a). This result also provided theoretical basis for further study of administration dose in rats.

When compared to urokinase, fibrinolytic circles occurred in both plasminogen-free fibrin plates and plasminogen-rich fibrin plates by the fibrinolytic activities of SK. In addition, the fibrinolytic circles in the latter plates were bigger than those in the later plates (Figure 4b).

Fibrinogen is composed of two sets of three polypeptide chains α, β and γ, which were joined by disulfide bridges at the N-terminal domain. To investigate the fibrinogenolytic activity of SK, the gel electrophoresis (SDS-PAGE) analysis was employed to study the degraded degree of chains α, β, and γ. As can be seen from Figure 4c, chain α, chain β, and chain γ of fibrinogen were degraded orderly, and the degree of degradation of three polypeptide chains were ranked in the order of chain α > chain β > chain γ, from the highest to the lowest. Additionally, three chains were nearly thoroughly degraded after reaction for 16 h.

### 2.5. Effects on FeCl_3_-Induced Common Carotid Arterial Thrombosis Rat of SK

We found significantly increased weight of thrombus in model group rats as compared with blank control group (*p* < 0.001) (Figure 4d). By administration of SK at different doses, when compared with model group, the weight of thrombus significantly was decreased and the inhibitory effect was equal to that of urokinase (*p* < 0.001) under low dose (1 μg/kg) treatment of SK. From morphological perspectives by hematoxylin-eosin staining (HE), with the increasing dose of SK, the thrombus of arteries was increasingly incompact, at the same time, media and intima of artery tend to be more complete. Moreover, clear outline, continuous intima, and little thrombus were even found in artery in the SKH group (Figure 4e).

According to the experimental results of four coagulation tests, the protective effects of SK on coagulation system were achieved through prolonging activation part thrombin time (APTT), which indicates intrinsic pathways, PT which indicates extrinsic pathways and thrombin time (TT) which reflects the time costs on conversion of fibrinogen into fibrous protein. When compared with control group, APTT, prothrombin time (PT), and TT sharply decreased and fibrinogen (FIB) increased in model group (*p* < 0.001). With the treatment of SK, these indexes markedly improved. In addition, the improved indexes of APTT, PT, TT, and FIB in SKM (10 μg/kg) and SKH (100 μg/kg) groups were basically better those of in urokinase group. (Table S2).

We found significantly increased weight of thrombus in model group rats compared with blank control group (*p* < 0.001) (Figure 4d). By the administration of SK at different doses, compared with model group, the weight of thrombus significantly was decreased and the inhibitory effect was equal to that of urokinase (*p* < 0.001) under low dose (1 μg/kg) treatment of SK. From morphological perspectives, the thrombus of arteries was increasingly incompact as well as media and intima of artery tend to be more complete with the increasing dose of SK. Moreover, clear outline, continuous intima, and little thrombus were even found in artery in SKH group (Figure 4e).

For further exploration of effect mechanism, the vascular regulatory factors (6-keto-prostaglandin F1α (6-keto-PGF1α), prostacyclin I_2_ (PGI_2_), and thromboxane B2 (TXB_2_)), fibrinolytic system regulatory factors (fibrin degradation products (FDP), plasminogen (PLG), plasminogen activator inhibitor-1 (PAI-1), and t-PA) and other regulatory factors (endothelin-1 (EF-1) and calcitonin gene-related peptide (CGRP)) were signally improved by SK at low (1 μg/kg), middle (10 μg/kg), and high (100 μg/kg) dose (*p* < 0.05, *p* < 0.01 or *p* < 0.001). Additionally, the improvement of the factors above in SK groups was much better than that of in urokinase group (Tables S3 and S4).

Untargeted mass spectrometry-based metabolomics profiling of antithrombotic effect mechanisms on FeCl_3_-induced common carotid arterial thrombosis in rats.

Based on the metabolomics method, the underlying mechanisms of SK were explored on FeCl_3_-induced common carotid artery thrombosis in rats by untargeted mass spectrometry. The metabolomics methodology was firstly validated by the quality control (QC) samples. The results indicated that the RSD% of repeatability was between 3.06% and 8.15% and RSD% of stability was between 4.35% and 7.35%, which was at 0, 2, 4, 8, 12, and 24 h during a single day.

Figure S1 showed representative based peak intensity (BPI) chromatograms of urine (A, B, C) and plasma (D, E, F). From the results, the obvious differences of chromatographic peaks were observed between sham-operated group and model group, as well as model group and SK group from different kinds of bio-samples (urine and plasma). In PCA scores and 3D-PLS-DA scores, good separation of samples was obviously presented between sham-operated group clusters and model group clusters, which suggested that a remarkable change was occurred in endogenous metabolism of rats from model group with arterial thrombus induced by FeCl_3_ (Figures S2A, S3A, S4A, S5A and S2B, S3B, S4B, S5B). Moreover, in the PLS-DA models established in current experiment, the R2Y and Q2 were 0.928 and 0.638 in positive mode and 0.947 and 0.761 in negative mode from urine samples, at the same time, they were 0.989 and 0.560 in positive mode and 0.952 and 0.678 in negative mode from plasma samples. The R2Y and Q2 of PLS-DA models from urine and plasma samples indicated that the analytical results were reliable for metabolomics studies. The common carotid artery thrombosis model induced by FeCl_3_ was verified to be successful. The variables played important roles in distinguishing sham-operated groups from model groups screened by VIP >1 in S–Plot of OPLS-DA and Loading–Plot of PLS–DA, which were regarded as potential biomarkers and they were studied for further confirmation (Figures S2C, S3C, S4C, S5C and S2D, S3D, S4D, S5D).

**Figure 5:**
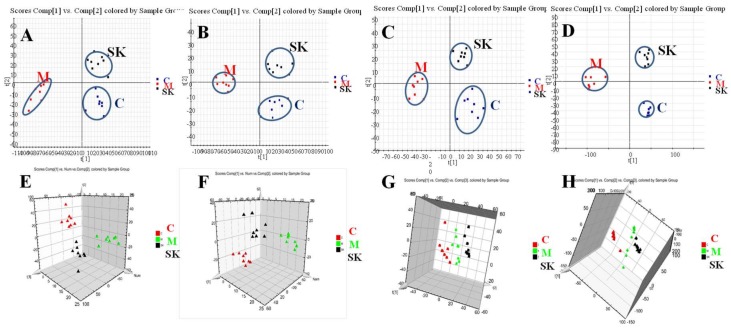
The PLS-DA score plot (**A**,**B**,**C**,**D**) and 3-D PLS-DA score plot (**E**,**F**,**G**,**H**) of urine samples (**A**,**B**,**E**,**F**) and plasma samples (**C**,**D**,**G**,**H**) from sham-operated, model and SK groups both in positive (**A**,**E**,**C**,**G**) and negative modes (**B**,**F**,**D**,**H**).

As shown in both positive and negative mode, the urine and plasma samples in three groups were signally clustered (Figure 5). Besides, when compared with model groups, the quantitation of relative distance was also showed that the relative distance between SK groups and model groups were observably shorter than that of between model groups and sham-operated groups (*p* < 0.01) (Table S5). It revealed that from the perspective of endogenous metabolites, SK had significant inhibitory effects on FeCl_3_-induced arterial thrombosis.

Finally, 9 biomarkers were identified from urine and plasma as leukotriene C4, thromboxane A2, dihydroceramide, tryptamine, sphinganine, arachidonic acid, niacinamide, phytosphingosine, and lysoPC(18:1(9Z)) (Table S6). The semiquantitative method was employed to display the content of the identified 9 biomarkers. When compared with sham-operated groups, the relative abundance of leukotriene C4, thromboxane A2, sphinganine, phytosphingosine, arachidonic acid, and lysoPC(18:1(9Z)) in the model group raised markedly (*p* < 0.01), while the relative abundance of dihydroceramide and tryptamine dropped significantly (*p* < 0.01). When compared with the model group, the relative abundance of leukotriene C4, thromboxane A2, sphinganine, phytosphingosine, arachidonic acid, and lysoPC(18:1(9Z)) in the SK group jumped markedly (*p* < 0.05 or 0.01), while the relative abundance of dihydroceramide and tryptamine dropped significantly (*p* < 0.01) (Table S7). The above results illustrated that the model of arterial thrombosis was successfully built in rats and SK could improve and treat the metabolic disorders in rats with arterial thrombosis.

**Figure 6:**
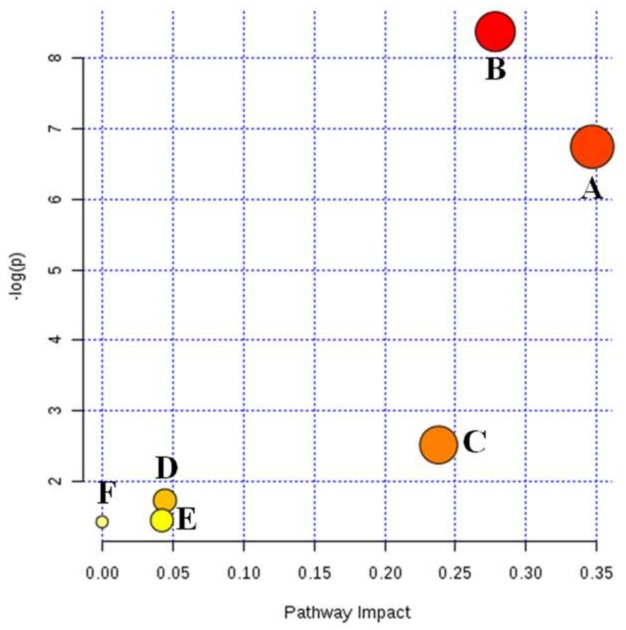
Metabolomics profiling of antithrombotic effect mechanisms on FeCl_3_-induced common carotid arterial thrombosis in rats. (A) arachidonic acid metabolism, (B) sphingolipid metabolism, (C) nicotinate and nicotinamide metabolism, (D) glycerophospholipid metabolism, (E) tryptophan metabolism, and (F) biosynthesis of unsaturated fatty acids.

The nine biomarkers were further imported into MetaboAnalyst 3.0 for ingenuity network analysis and six relevant metabolic pathways, including arachidonic acid metabolism, sphingolipid metabolism, nicotinate and nicotinamide metabolism, glycerophospholipid metabolism, tryptophan metabolism, and biosynthesis of unsaturated fatty acids were conducted to be mostly responsible for the common carotid arterial thrombosis (Figure 6). As shown in Table S8, arachidonic acid metabolism with an impact value of 0.3468, sphingolipid metabolism with an impact value of 0.2782, as well as nicotinate and nicotinamide metabolism with impact value of 0.2381 were regarded as three most important metabolic pathways in our experiments.

## 3. Discussion

Authors should discuss the results and how they can be interpreted in perspective of previous studies and of the working hypotheses. The findings and their implications should be discussed in the broadest context possible. Future research directions may also be highlighted.

In our study, after an efficient extract method, a fast and gentle (NH_4_)_2_SO_4_ graded precipitation method was applied to remove fat-soluble and other water-soluble constituents, including some protein, which could not only be beneficial to concentrate the extract volume, but also observably maintain the activity of SK.

Then in the following process of purification, a successional method involving a hydrophobic interaction chromatography, an ion exchange chromatography, and a gel filtration chromatography was applied and optimized in our study. Normally, according to Hofmeister series, (NH_4_)_2_SO_4_ was always chosen as a high salt phase in hydrophobic interaction separation. At high concentrations of (NH_4_)_2_SO_4_, hydrophobic ligands combined most of protein in column and removed other constituents of non-protein. Meanwhile, when the ratio of (NH_4_)_2_SO_4_ went down, the protein was eluted successively due to degressive interaction [19]. In this experiment, a Phenyl Sepharose High Performance column showed strong interaction with protein in condition of 1 mol/L (NH_4_)_2_SO_4_. Besides, we were fortunate enough to attain high recovery of SK since SK could be easily eluted from this column with deionized water, which also provided the possibility of high efficient recycling use of this column.

As a special biological macromolecule, protein is charged and is differently depended on its own isoelectric point (pI) and its surrounding pH. Based on this property, ion exchange chromatography is commonly used in protein purification based on the interaction of ion exchange ligands and target protein. According to law of chromatographic action, ion exchanger loads more protein under lower concentrate of saltiness, as the salinity rises, the binding protein is also eluted with the binding sites decreases [20,21]. The column of ion exchanger is employed to purify target protein on Q Sepharose High Performance. Proper pH is important parameter for purifying the charge of protein. Different pH values between 6.5 and 9.5 were investigated in preliminary tests. Consequently, the closest combination of protein and anion exchanger occurred at pH 8.5. Unfortunately, some chromoprotein in loading sample was always absorbed while using Q Sepharose High Performance as strong anion exchanger, which was not easily cleaned up in ion exchange chromatography. To overcome these defects, a flushing combination of 2 mol/L NaCl and 1 mol/L NaOH was used for making empty sites at one sample injection interval.

In the purification of protein, gel filtration chromatography exhibited irreplaceable value based on molecular size as well as mild separation conditions [22]. In this study, a Superdex G-75 gel filtration column with molecular range of 3000–70,000 Da was applied in the last step, which was used to further purify the protein. Besides during the purification, 3500 Da dialysis bags, Zeba spin desalting columns, and Millipore amicon ultra 3K ultrafiltration were also used for desalting, purification, and concentration of samples.

The protein contents and total enzyme activities of obtained fractions were determined for calculation of specific activity, yield and purity. The yield of fractions (total enzyme activity after each step/total enzyme activity after extraction × 100%) collected after all the steps utilized in our experiments was 14.47%, and the purification fold (specific activity after each step/specific activity after extraction × 100%) was 2466.62 (Table S9). Despite that we got relative high purity of SK in this study, we still have to admit that as process goes in preparation procedure, total activity of SK inevitably decreases. Thus, we still need to seek much more simplified and quick methods in future study if we aim at studying the activity and its mechanism of SK.

After the identification of purity by the methods of Native PAGE, SDS-PAGE and gel filtration chromatography, SK was further detected by MALDI-TOF for molecular weight determination. The molecular weight of SK was 28.0kDa, which was similar to many other fibrinolytic proteases, including nattokinase (27.3 kDa), urokinase (34.0 kDa), and the lumbrukinase in Eisenia foetida (24.0 kDa and 34.0 kDa) and Neanthes japonica (33.5 kDa) with anticoagulative activity [23]. It also came to reasonable agreement with the results determined by SDS–PAGE.

Because the biosynthesis of protein was normally started from N terminal, the posttranslational modification of N terminal usually possessed close relationship with its biological functions and stabilities [24]. Therefore, N terminal determination analysis was helpful for further analysis of higher spatial structures and for revealing functional mechanism of protein. Edman degradation method is a powerful method that is widely used in N-terminal amino acids sequencing [25]. With the help of Edman degradation, the 15 N-terminal amino acids sequence was PFPVPDPFVWDTSFQ, which showed 84% of identities with hemerythrin in *S. nudus*. When compared with many other peptides with antithrombotic activity or fibrinolytic activity, it was found that SK was a novel active peptide from the aspect of the N terminal amino acids including urokinase, lumbrukinase, nattokinase, streptokinase and other fibrinolytic enzymes (Table S10).

It was crucial to develop the research on the enzymatic properties for further intensive study and reasonable utilization on SK. The properties of SK showed the activity difference of SK had no significant differences and pH value, which meant that SK was a thermostable protease that is resistant to acids and alkalis in some degree. The effects on activities of SK by different metal ions showed not significantly different as well, however, according to obvious inhibitory effects by PMSF and aprotinin when compared with leupeptin and pepstatin, we could come to a conclusion that SK typically belonged to serine protease category.

The studies of in vitro fibrinolytic activities of SK showed that SK not only presented direct effects on fibrinogen, but also had indirect effects on fibrinogen by activating plasminogen. According to the result of SDS–PAGE, it was found that the fibrinolytic effects of SK were established by degrading chain α, chain β, and chain γ in fibrinogen to inhibit the form of fibrin. Hence, SK was a plasminogen activator with the capacity of direct fibrinolysis.

The thrombosis was usually formed from coagulation disorders that were induced by vascular endothelial cells injury or platelet aggregation as well as changes on the hemorheology [26]. To simulate the properties of thrombosis as much as possible, the FeCl_3_-induced common carotid artery thrombosis model with easy operation and highly successful rate was applied in our experiment. This method was widely used in many researches in regard to the occurrence and development of thrombosis as well as exploitation of therapy methods and drugs [27,28]. The idea was that when FeCl_3_ stimulated the vascular wall, the oxidative stress reacted by free radicals led to a series of reactions, including the lipid peroxidation, endothelial injury, platelet adhesion and aggregation, and finally the occluded thrombus [29]. During the formation of thrombus, a series of endogenous regulatory factors were also involved in the regulation. TXA_2_ and PGI_2_ were two important regulation substances from arachidonic acid (AA) metabolism with contrary function: TXA_2_ presented certain effects of contracting the vascular and of promoting the platelet to aggregate, adhere and release, while PGI_2_ was regarded as a powerful active substance with an antithrombotic effect, which had the ability of dilating the blood vessels, inhibiting the platelet aggregation, promoting fibrinolytic activity, and so on. These two substances were unstable in vivo and could be easily metabolized into TXB_2_ and 6-keto-PGF_1α_, respectively. Hence, these two metabolites were detected to assess the contents of TXA_2_ and PGI_2_ in most cases [30]. Tissue type plasminogen activator (t-PA) and urokinase type plasminogen activator (u-PA) could activate PLG into plasmin, which inhibited thrombus by catalyzing fibrin to degrade into fibrin degradation products (FDP). PAI-1 was the substances physiologically inhibit t-PA and u-PA [31]. Besides endothelin (ET-1) lying in vascular endothelial cells also had vital function in regulating cardiovascular efficiency and in maintaining basic vascular tension and cardiovascular homeostasis. As an endogenously long-acting vasoconstrictor, it kept long action without antagonism by α receptor antagonists, H1 receptor antagonists, and 5-HT receptor antagonists [32]. Finally, the CGRP was proved to be a bioactive polypeptide that regulated the cardiovascular system by relaxing blood vessel, strengthening myocardiac contraction, accelerating heart rate, dilating coronary artery, and so on [33].

By the measurement of weight of thrombus and observation of histomorphology, we found that SK had a significant therapeutic effect on carotid arterial thrombosis in rats, and SK got a distinctly better effect than that of urokinase in most cases. The exploration of underlying mechanisms revealed that the antithrombotic effect of SK, which was represented by inhibition of FeCl_3_-induced thrombosis in the common carotid arterial was related to the regulation of vascular, fibrinolytic, and coagulation systems.

Based on the mechanism study by in vivo experiments, the underlying mechanisms of SK on FeCl_3_-induced common carotid artery thrombosis in rats were further studied by untargeted mass spectrometry-based metabolomics profiling. Three classical metabolism pathways were discovered and responsible to illustrate the effect mechanisms.

AA metabolism pathway was an important metabolic pathway in occurrence and development of thrombotic disease. It acted as a metabolic substrate and also an important precursor of several bioactive substances [34]. During the process of thrombus development, the vascular endothelial cells promoted the conjunction type of AA to break into free AA by phospholipase A_2_ (PLA_2_) and released them out. Then, under the reducing action of the cyclo-oxygenase (CO), some free AA turned into prostaglandin G_2_ (PGG_2_) and further turned into prostaglandin H_2_ (PGH_2_). PGG_2_ and PGH_2_ were particularly unstable intermediate products, and they will further turn into other prostaglandins and thromboxanes, such as TXA_2_ and PGI_2_ [35]. Some free AAs turned into 5-HPETE by double oxidation of 5-lipoxygenase (5-LOX), and some of 5-HPETE further turned into 5-HETE by 5-LOX, while the other turned into leukotriene A_4_ (LTA_4_). LTA4 was an unstable intermediate of monooxygenase, and some LTA_4_ turned into leukotriene B_4_ (LTB_4_) by adding H_2_O or turned into leukotriene C_4_ (LTC_4_) by glutathione S-transferase. LTC_4_ possessed the certain effects of a contracting blood vessel, which was equal to angiotensin and even stronger than LTD_4_ and LTE_4_ [36,37]. In the current study, the contents of AA, LTC_4_, TXA_2_ and TXB_2_ were enhanced in FeCl_3_-induced common carotid arterial thrombosis rats. The results indicated that thrombus was easily excitated when the contents of AA, LTC_4_, TXA_2_, and TXB_2_ were raised in organism. However, these endogenous substances (such as AA, LTC_4_, TXA_2_, and TXB_2_) were significantly improved in model rats with the administration of SK. Based on the method of metabolism, SK exerted a key role in inhibiting vessel contraction, platelet aggregation, adhesion, and release by adjusting the AA metabolism pathway (Figure S6).

Recently, more and more researches focused on the disorders of sphingolipid metabolism, which leads to a disorder of signal transduction and even a series of metabolic diseases. In sphingolipid metabolism pathway, ceramides were the center substances of sphingolipid biosynthesis, degradation, and metabolism, as well as the general precursors of further sphingolipid metabolism [38]. Some studies have found that the contents of ceramide rose significantly in the endothelial cell dysfunction, and ceramides might mediate the metabolic dysfunction of endothelial cells [39]. There were also some studies discovered in the case of vascular injury, a vascular endothelial growth factor (VEGF) that is specific to vascular endothelial cells would mediate angiogenesis, and it also leads to a sharp rise in glycosphingolipid (a combination of ceramides and carbohydrate chain). In the latter studies, although the specific mechanism is not fully clear, some hypotheses suggested that sphingolipid metabolism might be involved in the regulation of VEGF [40,41]. All of the above results indicated that ceramides accumulation was closely related to endothelial cell dysfunction. In our research, the marked increase of dihydroceramide, which was an important precursor of ceramides, indicated that SK might play positive regulation on endothelial cell dysfunction by compensating the contents of dihydroceramide (Figure S7).

In the process of thrombosis, as oxidization-reduction coenzymes, nicotinamide adenine dinucleotides (NAD) and nicotinamide adenine dinucleotide phosphate (NADP) were usually served as electron acceptors and hydrogen donor in cells. Since they were synthesized by nicotinamides and ribose-adenine RNA, this reaction also caused the compensatory increases of nicotinic acids and nicotinamides [42]. When compared with the common carotid arterial thrombosis model group, the contents of niacinamide, which could be metabolized by NAD, NADP, nicotinamide ribotide, and nicotinamide riboside notably decreased in the SK group, which could imply that the process of thrombosis could be markedly slowed down with the function of SK (Figure S8).

The above results suggested that AA, sphingolipid, nicotinate, and nicotinamide mechanism pathways have close relationship with thrombus development. SK could significantly improve the metabolic abnormalities in common carotid arterial thrombosis of rats. The untargeted mass spectrometry-based metabolomics profiling of the thrombosis feature and underlying mechanisms of SK on thrombosis were firstly constructed. This study provided a systematic view of the development and progress of common carotid arterial thrombosis, and it also offered the application of evaluating the mechanisms of SK on common carotid arterial thrombosis induced by FeCl_3_ and other thrombotic diseases.

In summary, a set of methods was first established in the study, including freeze-thaw coupled with homogenate, (NH_4_)_2_SO_4_ graded precipitation, and combination of hydrophobic interaction chromatography, anion-exchange chromatography, and gel filtration chromatography for extraction, isolation, and purification of the thrombolytic protease in *S. nudus* based on the fibrinolytic activities tracking by fibrin plate assay. This chromatographically and electrophoretically pure peptide with a molecular weight of 28,003.67 Da and a 15 N-terminal amino acids sequence of PFPVPDPFVWDTSFQ was found to be a novel active peptide when comparing with other antithrombotic and fibrinolytic peptides.

## 4. Materials and Methods

### 4.1. Sources of S. Nudus Samples

*S. nudus* samples were collected and identified by Shao-Xiong Ding (Xiamen University, Xiamen, China). The samples were kept in −80 °C refrigerators after collection.

### 4.2. Animals

Forty-eight male Sprague-Dawley (SD) rats (220–250 g) were obtained from Experimental Animal Center of Zhejiang Province (SCXK (2016-0001), 10 January 2016). The rats were kept in plastic cages with fresh water and pellet food. The environmental temperature and humidity were respectively maintained at 24 ± 2 °C and 40–60%. Animal welfare and experimental procedures were carried out in accordance with the guide for care and use of laboratory animals. The ethic approval number was ACU-25 (20161229).and the approval date was 29 December 2016.

### 4.3. Sample Preparation

Fresh samples (800 g) were carefully dissected and removed excreta and 1600 mL of PBS was added for homogenization (1000 r/min, 30 s). Then, the homogenate was extracted by freezing and thawing twice. Finally, extract of *S. nudus* was obtained by collecting and mixing two-step supernatant.

### 4.4. Protease Isolation and Purification

The best range of (NH_4_)_2_SO_4_ percent saturation was chosen for preparing SK according to fibrinolytic activities that were determined by fibrin plate assay. The details of choosing best range of (NH_4_)_2_SO_4_ was described in “1. Option of best best range of (NH_4_)_2_SO_4_ in precipitation” in Supplementary Materials.

ÄKTA™ pure system (GE Health, Chicago, MA, USA) was employed to purify SK. SK was first isolated with hydrophobic interaction chromatography by fractionating extract through Phenyl Sepharose High Performance column (26 mm × 170 mm, GE Healthcare, Chicago, MA, USA). The mobile phase A and mobile phase B were PBS buffer and 1 mol/L (NH_4_)_2_SO_4_ solution. The flow rate was 3 mL/min. The linear gradient conditions were: 100% B, 3CV; 100–60% B, 0.5 CV; 60% B, 0.75 CV; 60–40% B, 0.25 CV; 40% B, 0.75 CV; 40–20% B, 0.25 CV; 20% B, 0.75 CV; 20–0% B, 0.25 CV; 0% B, 2 CV. The collected volume was set at 9 mL per fraction and the fibrinolytic activity to protein content ratio (FA/PC) of each fraction was monitored by UV detector at the wavelength of 280 nm. The collected fractions with high value of FA/PC were pooled and then desalted.

The sample purified with above method was loaded onto a Q Sepharose High Performance ion-exchange column (26 mm × 180 mm, GE Healthcare) with a gradient elution by phase A (PBS buffer) and phase B (0.8 mol/L NaCl). The flow rate was set at 3 mL/min and the system was eluted as following: 0% B, 2 CV; 0−20% B, 0.25 CV; 20% B, 2 CV; 20−30% B, 0.25 CV; 30% B, 1 CV; 30−50% B, 0.25 CV; 50% B, 1 CV; 50−70% B, 0.25 CV; 70% B, 0.75 CV; 70−100% B, 0.25 CV; 100% B, 2 CV. The samples were collected with the same method described above.

After the above process of isolation and purification, the sample was ultimately loaded onto the gel filtration column of Superdex prep grade G-75 (16 mm × 500 mm, GE Healthcare), equilibrated with PBS buffer of an isocratic elution at a flow rate of 0.8 mL/min. Finally, the protease SK was obtained followed by the collection method described above.

### 4.5. Identification of Purity, Determination of Molecular Weight and Analysis of N-terminal Amino Acids Sequence

SDS–PAGE, Native–PAGE and gel filtration chromatography were employed to identify the purity of SK and the methods were displayed in items “SDS-PAGE and Native PAGE of SK” and “Gel filtration chromatography of SK” in supporting information. Besides, molecular weight and N-terminal amino acids sequence of SK was respectively determined and analysed as the details in items “Determination of molecular weight” and “Analysis of N-terminal amino acids”.

### 4.6. Enzymatic Properties

In this experiment, the enzymatic properties of SK were studied from four aspects, including temperatures, pH values, metal ions, and inhibitors. The effects of temperatures on SK were studied by determining the residual activities by fibrin plate method after SK (20 μg/mL) pre-incubated at different temperatures (10, 20, 30, 40, 50, and 60 °C) for 24 h. The effects of pH on SK were researched by determining the residual activities after SK was being dissolved in different buffer solution with a series of pH values (pH = 4.0, 5.0, 6.0, 7.0, 8.0, 9.0, 10.0, and 11.0). In order to explore the effects of metal ions (K^+^, Na^+^, Mg^2+^, Ca^2+^, Cu^2+^, Fe^2+^, Zn^2+^, Al^3+^, Fe^3+^, and Mn^2+^) and protease inhibitors (ethylenediamine-tetraacetic acid (EDTA), phenyl methyl sulfonyl fluoride (PMSF), aprotinin, leupeptin, and pepstatin A) on SK, it was pre-incubated with different metal ions and different protease inhibitors respectively, and then the residual activities were determined for evaluating SK properties.

### 4.7. Fibrinolytic Activity and Fibrinogenolytic Activity

To study the fibrinolytic activity of SK on the degradation of fibrin, the fibrinolytic activities of SK in different concentrations (100.00, 50.00, 25.00, 12.50, 6.25, 3.06, and 1.53 μg/mL) were respectively determined by fibrin plate assay.

For further exploration of the fibrinolytic mechanism of SK, one fibrin plate was heated at 85 °C for 30 min in advance to inactivate plasminogen, and another plate without any treatment was served as control. Aliquots (10 μL) of 2000 IU/mL urokinase and 10 μg/mL SK were added into two plates, respectively.

Fibrinogenolytic mechanism of SK was further studied based on the above researches. Ten aliquots (200 μL) of 1% fibrinogen were mixed with 50 μL of 10 μg/mL SK and then the reaction solutions were respectively incubated at 37 °C for 0, 5, 10, 30 min, 1, 2, 4, 6, 8, and 16 h. After cooling down, aliquots of the reaction solutions (20 μL) were loaded onto SDS-PAGE consisting of 12% separation gel and 5% stacking gel for analysis.

### 4.8. Antithrombotic Effect on FeCl_3_-Induced Common Carotid Arterial Thrombosis in Rats

After one week of acclimatization, 48 rats were randomly divided into six groups: sham-operated group (C), FeCl_3_-induced common carotid arterial thrombosis model group (M), urokinase group (UK, 2000 IU/kg), high-dose SK group (SKH, 100 μg/kg), middle-dose SK group (SKM, 10 μg/kg), and low-dose SK group (SKL, 1 μg/kg). The rats in groups UK, SKH, SKM and SKL were injected with corresponding medication by caudal vein continuously for seven days; meanwhile, the groups C and M were injected with sterile water. On the seventh day, the FeCl_3_-induced common carotid arterial thrombosis was modeled in rats in groups UK, SK, and M (see item “Establishment of FeCl_3_-induced common carotid arterial thrombosis” in supporting information) and rats in group C was set as control. Then the blood samples (approximate 8 mL) were collected by abdominal aorta intubation approach in rats and divided into two parts. One part of 6 mL blood (3.8% sodium citrate as anticoagulant) was centrifuged (3000 r/min, 10 min) to get plasma for four coagulation tests (activation part thrombin time (APTT), thrombin time (TT), prothrombin time (PT), fibrinogen (FIB)), and four Elisa kit tests (thromboxane B2 (TXB_2_), plasminogen activator inhibitor-1 (PAI-1), plasminogen (PLG), and tissue t-PA. Another part of 2 mL blood (1.5% EDTA as anticoagulant) was centrifuged (3000 r/min, 10 min) to get plasma for five Elisa kit tests (calcitonin gene-related peptide (CGRP), endothelin-1 (ET-1), fibrin degradation products (FDP), 6-keto-prostaglandin F1α (6-keto-PGF1α), and prostacyclin I_2_ (PGI_2_). The arterial thrombus on the right artery (A) as well as segments with equal length (B) on the left artery of all rats executed were quickly cut off and respectively weighed for calculating thrombus weight (A–B). After weighing, the arterial thrombus was fixed in formalin and was embedded by paraffin for cutting sections and HE staining.

### 4.9. Mechanism of Effect on FeCl_3_-Induced Common Carotid Arterial Thrombosis Based on Metabonomics

To further investigate the mechanism of effect, an untargeted metabonomics method was established. The detail method was described in Supplementary Materials.

## 5. Conclusions

The study of properties showed that SK was a thermostable protease that is resistant to acids and alkalis in some degree. The effects on activities of SK by different metal ions showed no significant differences as well, however, according to obvious inhibitory effects by PMSF and aprotinin when compared with leupeptin and pepstatin, it could come to a conclusion that SK typically belonged to serine protease category. The in vitro fibrinolytic activities of SK by fibrin plate assay indicated that SK not only presented direct effects on fibrinogen but it also had indirect effects on fibrinogen by activating plasminogen, and the gel electrophoresis analysis results of fibrinogenolytic mechanism showed that the degradation of subunit in fibrinogen was in the order: chain α, chain β, and chain γ, and three chains were nearly thoroughly degraded after 16 h. By means of FeCl_3_-induced carotid arterial thrombosis in rats, SK was found to possess anapparently better antithrombotic effect than that of urokinase related to regulation of vascular, fibrinolytic and coagulation systems. Moreover, the untargeted mass spectrometry-based metabolomics profiling of thrombosis feature and underlying mechanisms of SK on thrombosis were also first completed, and it not only provided a systematic view of the development and progress of common carotid arterial thrombosis, but also offered mechanistic insights for SK on common carotid arterial thrombosis induced by FeCl_3_ by inhibiting vessel contraction, platelet aggregation, adhesion, and release, improving endothelial cell dysfunction and weakening thrombus formation process.

## Supplementary Materials

Supplementary materials can be found at http://www.mdpi.com/1422-0067/19/10/3023/s1.
