# Supplementary material for: A Novel Antithrombotic Protease from Marine Worm Sipunculus Nudus
Source: Int J Mol Sci. 2018 Oct 4;19(10):3023. doi: 10.3390/ijms19103023 (PMC6213608; doi:10.3390/ijms19103023)
Supplement: Supplementary file 1 [file ijms-19-03023-s001.pdf]

Table S1. Contents of samples during sample preparation.

Lyophilized Extract		Treated Worm		Fresh Worm (g)
Dry Weight (g)	Yield (%)	Wet Weight (g)	Yield (%)	
39.98	4.00	822.31	82.27	999.53

Table S2. Effects of SK on the four blood coagulation indexes of common carotid arterial thrombosis rats ($\overline{x} \pm S$, $n = 8$).

Group	APTT (s)	PT (s)	TT (s)	FIB (g/L)
C	50.59 ± 2.07	16.20 ± 0.63	30.72 ± 1.90	2.81 ± 0.20
M	30.12 ± 2.40 ***	8.67 ± 1.11 ***	21.46 ± 2.02 **	4.74 ± 0.32 ***
UK	46.51 ± 2.53 ##	13.22 ± 1.08 ##	25.74 ± 1.36 #	3.75 ± 0.26 #
SKL	39.87 ± 2.04 ##	11.81 ± 0.87 #	25.85 ± 1.29 #	3.75 ± 0.11 ##
SKM	47.92 ± 1.83 ###	14.47 ± 1.18 ##	27.35 ± 1.15 #	3.48 ± 0.16 ##
SKH	49.46 ± 1.64 ###	15.69 ± 1.22 ##	28.68 ± 0.98 ##	3.20 ± 0.07 ##

* $p < 0.05$, ** $p < 0.01$, *** $p < 0.001$ vs. Sham-operated group; # $p < 0.05$, ## $p < 0.01$, ### $p < 0.001$ vs. Model group.

Table S3. Effects of SK on the contents of TXB_2, PAI-1, PLG and tPA of common carotid arterial thrombosis rats ($\overline{x} \pm S$, $n = 8$).

Group	TXB_2 (ng/L)	PAI-1 (pg/mL)	PLG (mIU/L)	tPA (ng/mL)
C	103.98 ± 5.52	64.31 ± 2.37	225.49 ± 2.35	11.05 ± 0.28
M	142.68 ± 6.65 **	81.38 ± 2.64 **	183.90 ± 4.17 ***	4.93 ± 0.47 ***
UK	121.96 ± 6.62 #	68.33 ± 0.96 ##	211.25 ± 4.95 ##	9.18 ± 0.17 ###
SKL	125.93 ± 6.79 #	69.54 ± 0.80 ##	216.90 ± 3.45 ###	9.17 ± 0.26 ###
SKM	118.84 ± 3.23 ##	66.17 ± 1.04 ###	221.12 ± 3.44 ###	9.71 ± 0.32 ###
SKH	110.35 ± 6.06 ##	64.43 ± 1.16 ###	229.01 ± 2.21 ###	10.77 ± 0.48 ###

* $p < 0.05$, ** $p < 0.01$, *** $p < 0.001$ vs. Sham-operated group; # $p < 0.05$, ## $p < 0.01$, ### $p < 0.001$ vs. Model group.

Table S4. Effects of SK on the contents of CGRP, ET-1, FDP, 6-keto-PGF1α and PGI_2 of common carotid arterial thrombosis rats ($\overline{x} \pm S$, $n = 8$).

Group	CGRP (ng/L)	ET-1 (μg/L)	FDP (ng/mL)	6-keto-PGF1α (ng/mL)	PGI_2 (ng/L)
C	108.66 ± 1.40	119.02 ± 3.57	59.93 ± 2.60	9.29 ± 0.46	177.47 ± 4.55
M	77.75 ± 3.16 ***	193.54 ± 4.89 ***	77.56 ± 2.42 ***	17.28 ± 0.66 ***	123.10 ± 6.16 **
UK	92.13 ± 1.12 ##	172.26 ± 4.22 ##	66.55 ± 1.44 ##	11.54 ± 0.52 ###	170.39 ± 2.53 ###
SKL	94.55 ± 2.61 ##	171.25 ± 2.30 ##	66.25 ± 1.62 ##	11.08 ± 0.90 ###	166.62 ± 2.63 ###
SKM	103.14 ± 4.45 ##	139.95 ± 8.08 ###	62.35 ± 1.10 ###	9.94 ± 0.15 ###	173.62 ± 1.76 ###
SKH	112.69 ± 2.44 ###	116.86 ± 5.98 ###	58.40 ± 1.30 ###	9.03 ± 0.06 ###	180.45 ± 2.50 ###

* $p < 0.05$, ** $p < 0.01$, *** $p < 0.001$ vs. Sham-operated group; # $p < 0.05$, ## $p < 0.01$, ### $p < 0.001$ vs. Model group

Table S5. The relative distance between the model groups as well as SK groups and sham-operated groups from the PLS–DA score plot of the urine and plasma samples ($\overline{x} \pm S$, $n = 8$).

Sample	ESI mode	Sham-operated		Model	SK
		X-axis	Y-axis		
Urine	+	20.20	−18.14	65.76 ± 6.73	46.28 ± 5.05 ##
	−	34.86	−31.65	108.21 ± 11.17	65.57 ± 7.36 ##
Plasma	+	22.62	−17.54	60.28 ± 4.97	39.89 ± 4.68 ##

–	45.32	−32.33	118.58 ± 9.24	61.34 ± 8.48 ##

# $p < 0.05$, ## $p < 0.01$, ### $p < 0.001$ vs. Model group.

Table S6. Potential biomarkers of the plasma and urine from common carotid arterial thrombosis rats and their identification results.

NO	Sample Source	VIP	Retention Time/min	Molecular Formula	M/Z	Metabolites	ESI Mode	HMDB ID	Structure
Um1	urine	2.66	4.33	$C_{30}H_{47}N_3O_9S$	625.7740	Leukotriene C4	+	01198	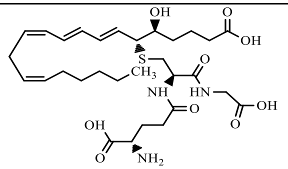
Um2	urine	4.87	10.44	$C_{20}H_{32}O_5$	352.2781	Thromboxane A2	+	01452	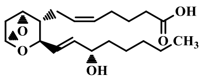
Um3	urine	3.62	10.44	$C_{19}H_{39}NO_3$	352.2781	Dihydroceramide	+	06752	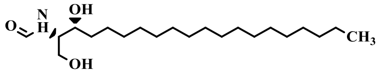
Um4	urine	4.12	4.34	$C_{10}H_{12}N_2$	162.0541	Tryptamine	+	00303	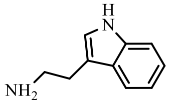
Um5	urine	4.78	11.93	$C_{18}H_{39}NO_2$	282.2772	Sphinganine	−	00269	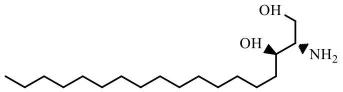
Um6	urine	4.70	12.37	$C_{20}H_{32}O_2$	339.2715	Arachidonic acid	−	01043	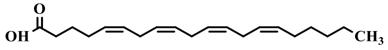
Um7	urine	3.30	9.00	$C_6H_6N_2O$	121.0646	Niacinamide	−	01406	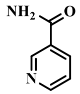
Pm1	plasma	6.54	11.64	$C_{18}H_{39}NO_3$	339.3458	Phytosphingosine	+	04610	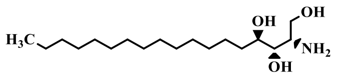
Pm2	plasma	5.21	7.54	$C_{26}H_{52}NO_7P$	544.3423	LysoPC(18:1(9Z))	+	02815	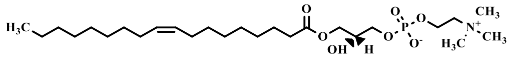

Table S7. The relative intensity changes of the potential biomarkers in different groups ($\overline{x} \pm S$, n = 8).

No	Metabolites	Sham-Operated	Model	SK
Um1	Leukotriene C_4	66.12 ± 4.21	92.83 ± 5.17 **	84.88 ± 1.49 $^{\#}$
Um2	Thromboxane A_2	51.74 ± 3.69	79.68 ± 2.51 **	68.45 ± 2.97 $^{\#\#}$
Um3	Dihydroceramide	172.47 ± 2.92	143.45 ± 2.07 **	163.47 ± 4.39 $^{\#\#}$
Um4	Tryptamine	97.06 ± 3.54	70.19 ± 3.43 **	86.29 ± 1.91 $^{\#\#}$
Um5	Sphinganine	59.56 ± 4.47	97.08 ± 2.31 **	77.58 ± 3.00 $^{\#\#}$
Um6	Arachidonic acid	155.66 ± 3.58	203.44 ± 9.14 **	191.66 ± 3.16 $^{\#}$
Um7	Niacinamide	118.67 ± 4.39	177.41 ± 6.20 **	139.11 ± 5.10 $^{\#\#}$
Pm1	Phytosphingosine	236.29 ± 6.65	326.62 ± 7.42 **	276.27 ± 4.89 $^{\#\#}$
Pm2	LysoPC(18:1(9Z))	351.16 ± 10.51	420.45 ± 8.98 **	377.37 ± 8.25 $^{\#\#}$

* $p < 0.05$, ** $p < 0.01$ vs. Sham-operated group; $^{\#}$ $p < 0.05$, $^{\#\#}$ $p < 0.01$ vs. Model group.

Table S8. Metabolomic pathways of the urine and plasma samples from common carotid arterial thrombosis rats.

Pathway	Total	Hit	Raw p	Holm p	Impact
Arachidonic acid metabolism	36	3	0.0017	0.13	0.3468
Sphingolipid metabolism	21	3	0.0003	0.03	0.2782
Nicotinate and nicotinamide metabolism	13	1	0.0892	1.00	0.2381
Glycerophospholipid metabolism	30	1	0.1951	1.00	0.0444
Tryptophan metabolism	41	1	0.2575	1.00	00423
Biosynthesis of unsaturated fatty acids	42	1	0.2630	1.00	0.0000

"Total" means number of compounds in pathways; "Hit" means matching number of biomarkers uploaded; Raw p value was calculated by pathway analysis; Impact value was calculated by topology analysis.

Table S9. Summary list of extraction, isolation and purification of SK.

	Total Protein Content (mg)	Total Enzyme Activity (IU)	Specific Activity (IU/mg)	Yield (%)	Purification Fold
Extract	5797.10	334260.79	57.66	100.00	1.00
$(NH4)_2SO_4$ graded precipitation	1897.08	251590.75	132.62	75.27	2.30
Hydrophobic interaction chromatography	52.54	182100.62	3465.94	54.48	60.11
Ion exchange chromatography	1.28	93770.47	73258.18	28.05	1270.52
Gel filtration chromatography	0.34	48356.61	142225.31	14.47	2466.62

Table S10. Comparison of N-terminal amino acid sequence of SK with other peptides.

Entry	Accession	Enzyme	Source	Matching	Similarity
1	AAA61252	urokinase	*Homo sapiens*	PFPVPDPFVWDTSFQ	0
2	AAL28118	lumbrukinase	*Lumbricus bimastus*	PFPVPDPFVWDTSFQ	0
3	AAW27919	lumbrukinase	*Eisenia fetida*	PFPVPDPFVWDTSFQ	0
4	ABM97611	nattokinase	*Bacillus subtilis*	PFPVPDPFVWDTSFQ	0
5	CAA51351	streptokinase	*Streptococcus dysgalactiae subsp. equisimilis*	PFPVPDPFVWDTSFQ	0
6	P86330	NJF	*Neanthes japonica*	PFPVPDPFVWDTSFQ	0
7	AGM38918	Fibrinolytic protein III	*Urechis unicinctus*	PFPVPDPFVWDTSFQ	0
8	AGS48987	fibrinolytic enzyme	*Arenicola cristata*	PFPVPDPFVWDTSFQ	13.33%
9	CAP08291	hemerythrin	*Sipunculus nudus*	PFPVPDPFVWDTSFQ	73.33%

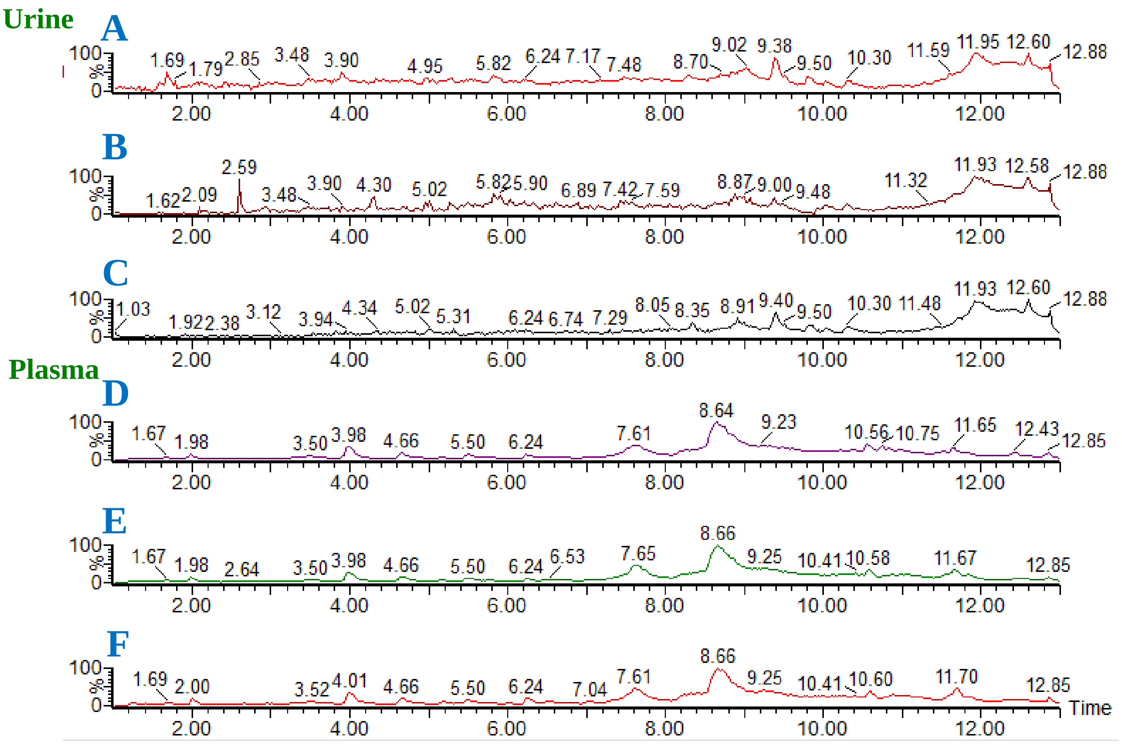

Figure S1. Representative BPI chromatograms of urine samples (**A**, **B**, **C**)and plasma samples(**D**, **E**, **F**) from sham-operated group (**A**, **D**), model group (**B**, **E**) and SK group (**C**, **F**).

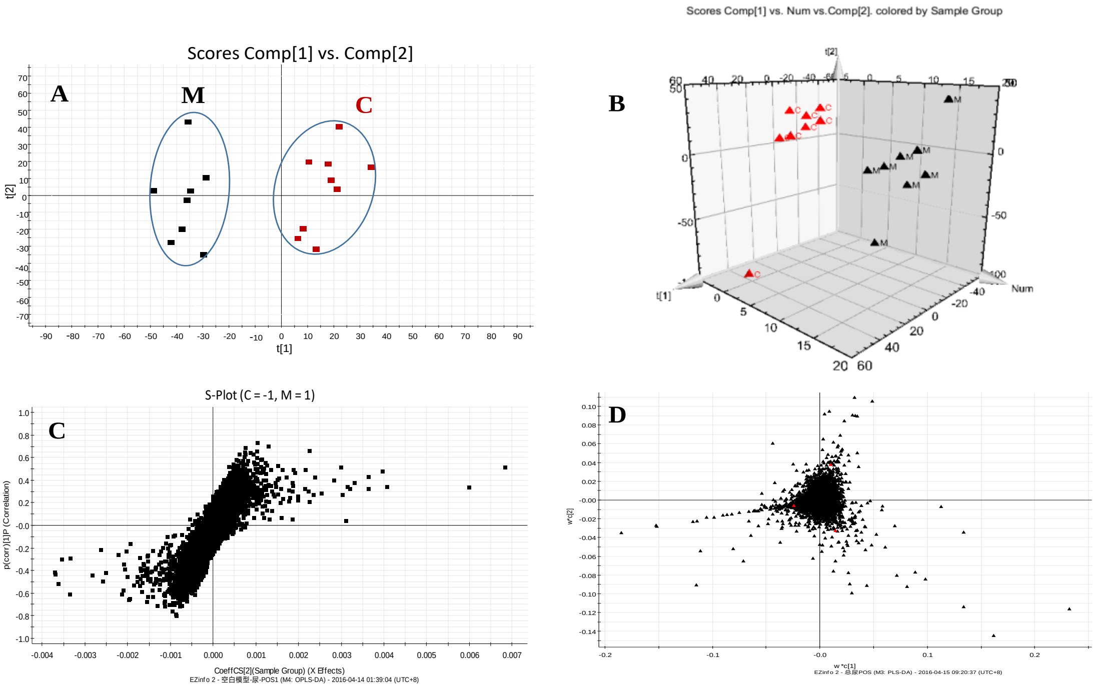

Figure S2. The PCA score plot (**A**), 3-D PLS–DA score plot (**B**), S-plot of OPLS–DA (**C**) and loading plot of PLS–DA (**D**) of urine samples from sham-operated and model groups in positive mode.

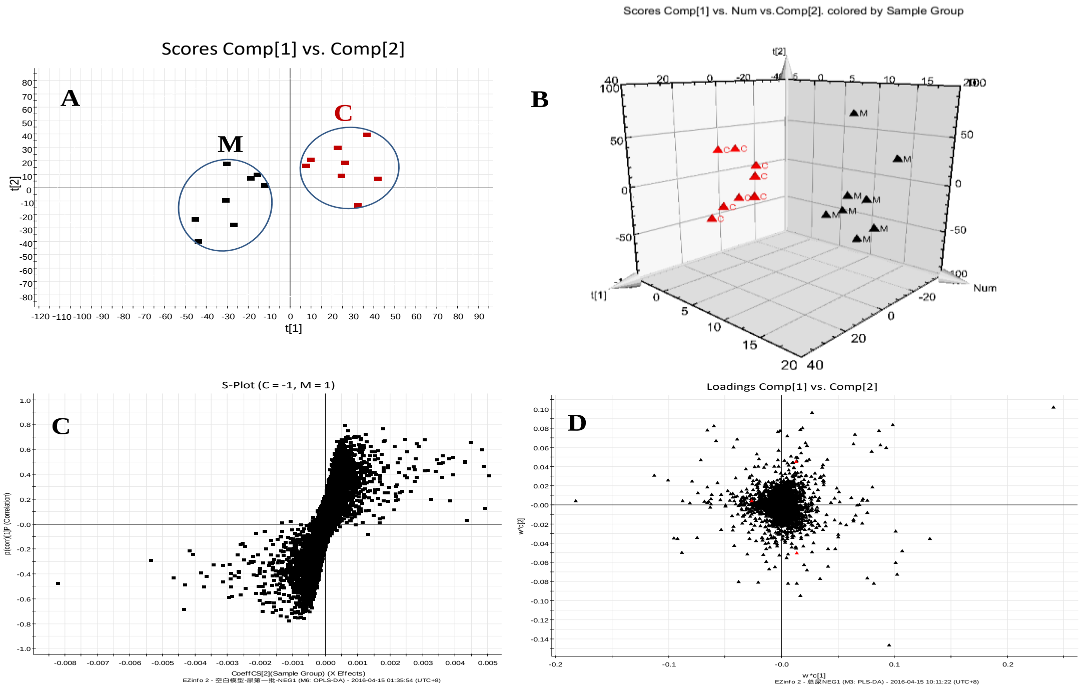

Figure S3. The PCA score plot (**A**), 3-D PLS–DA score plot (**B**), S-plot of OPLS–DA (**C**) and loading plot of PLS–DA (**D**) of urine samples from sham-operated and model groups in negative mode.

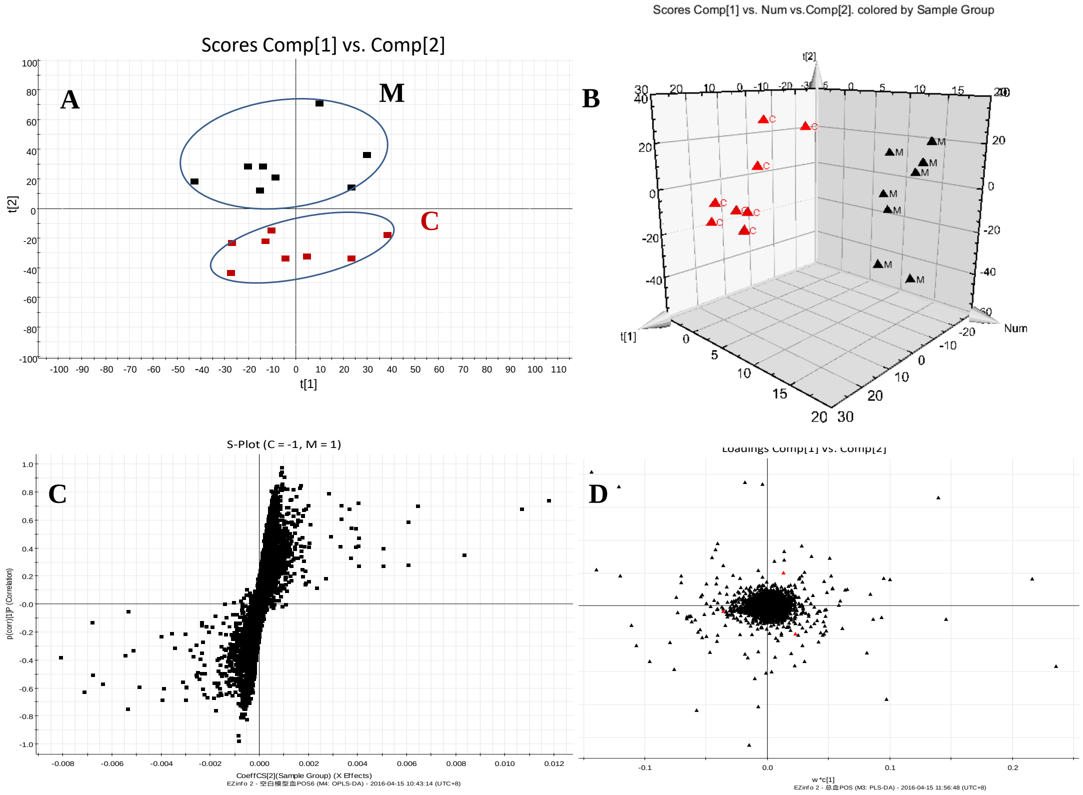

Figure S4. The PCA score plot (**A**), 3-D PLS–DA score plot (**B**), S-plot of OPLS–DA (**C**) and loading plot of PLS–DA (**D**) of plasma samples from sham-operated and model groups in positive mode.

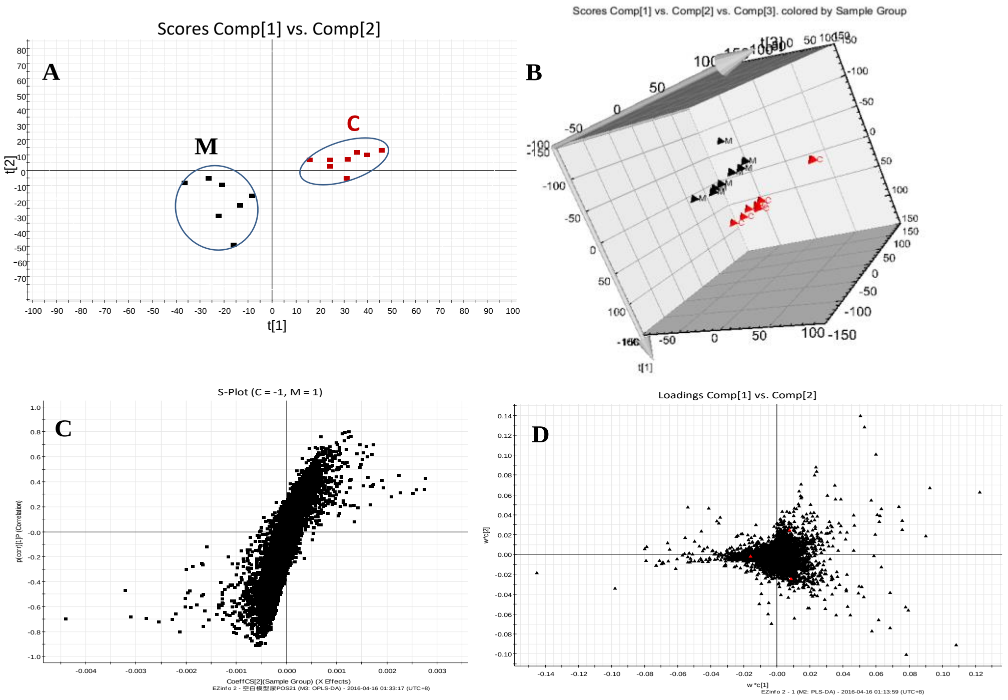

Figure S5. The PCA score plot (**A**), 3-D PLS–DA score plot (**B**), S-plot of OPLS–DA (**C**) and loading plot of PLS–DA (**D**) of plasma samples from sham-operated and model groups in negative mode.

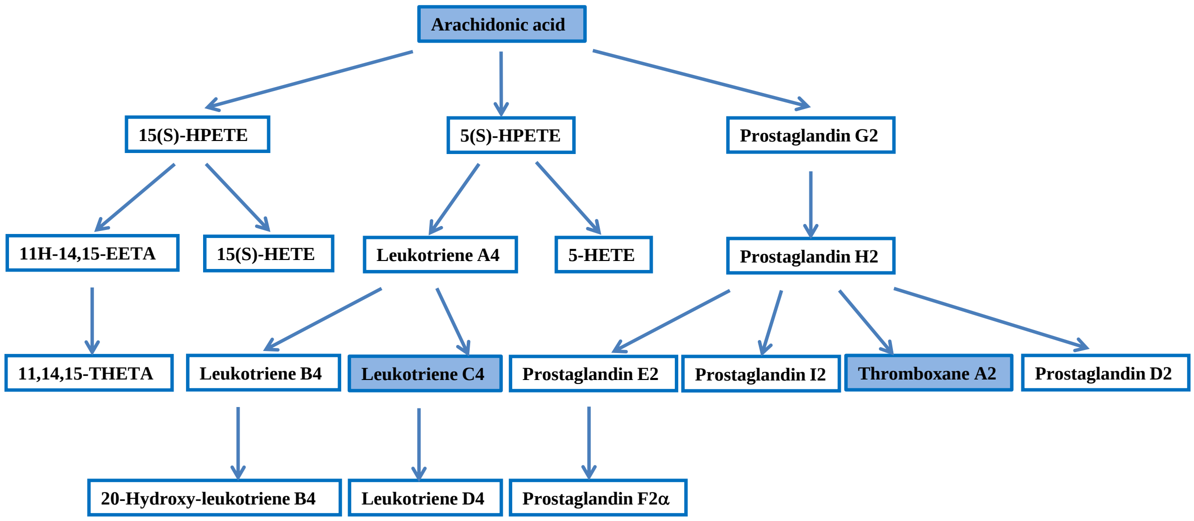

Figure S6. Construction of arachidonic acid metabolism pathways in rats.

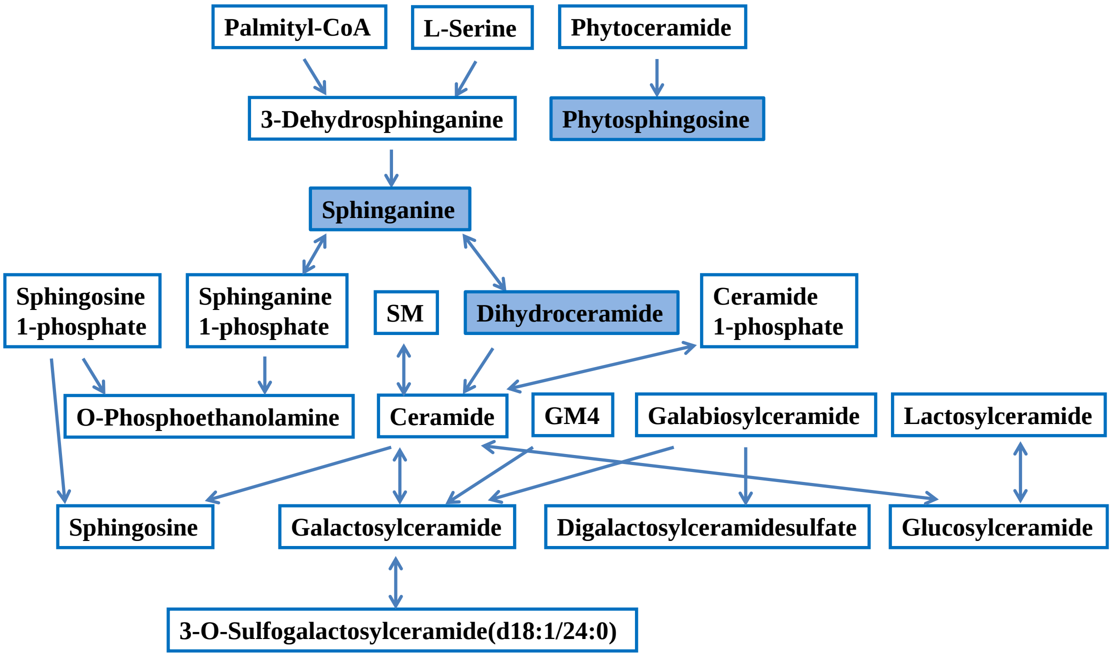

Figure S7. Construction of nicotinate and nicotinamide metabolism pathways in rats.

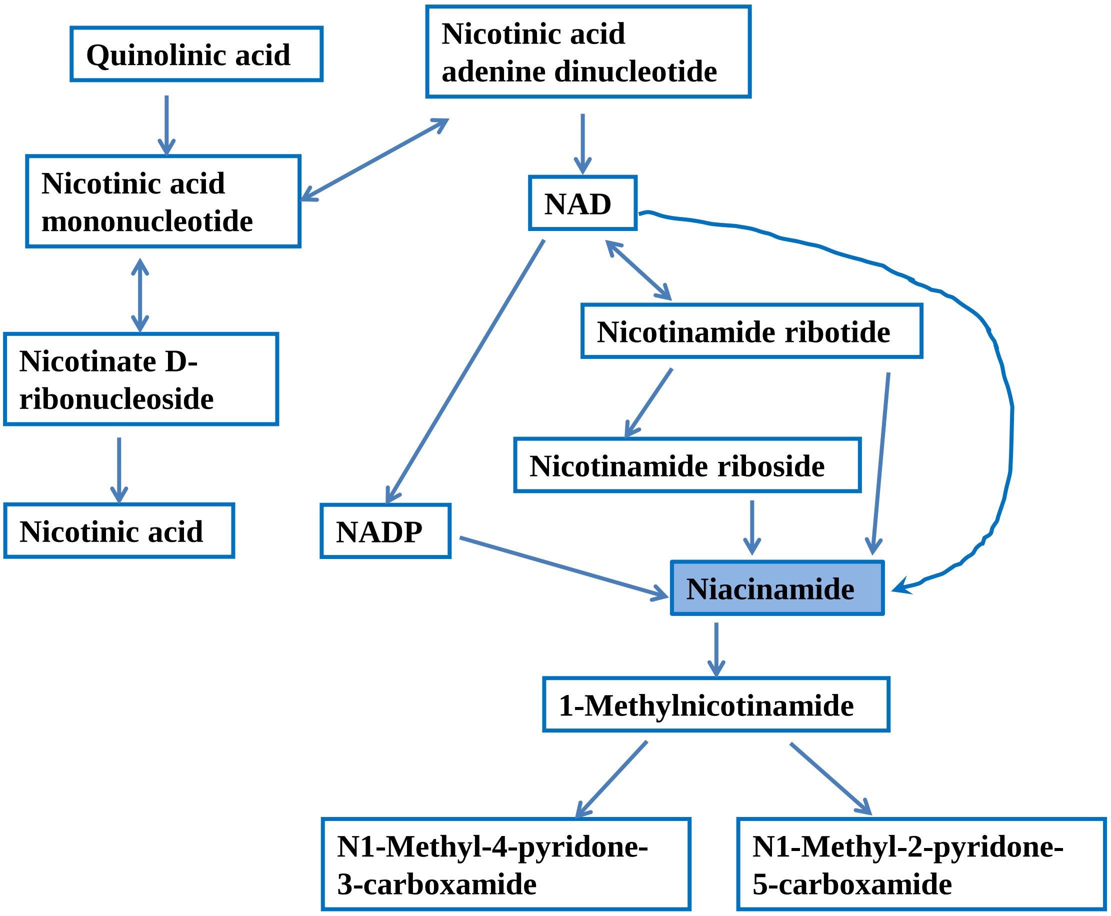

Figure S8. Construction of sphingolipid metabolism pathways in rats.

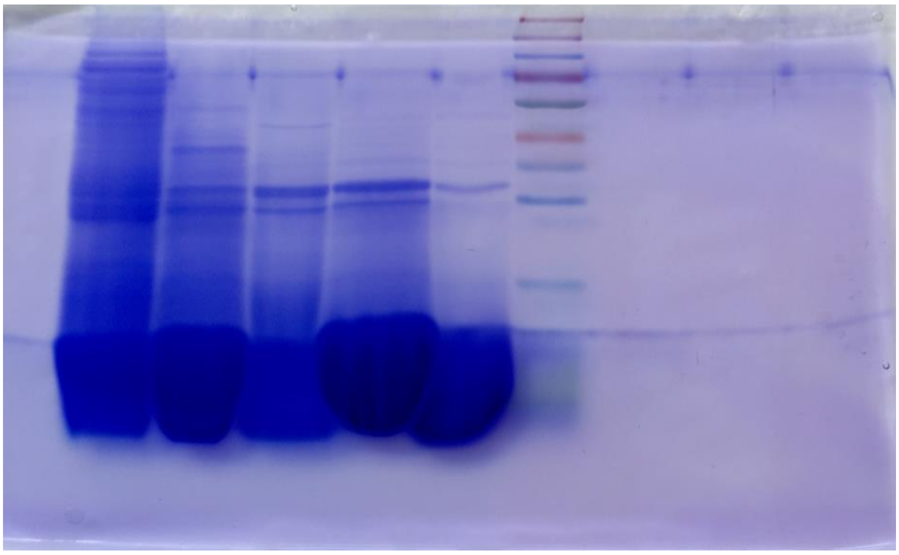

Figure S9. The full-length gel of electronic analysis of SDS-PAGE for different steps.
